# Association of anti-catecholaminergic antiarrhythmic drugs with survival in sepsis-associated new-onset atrial fibrillation

**DOI:** 10.1038/s41598-026-51249-9

**Published:** 2026-07-06

**Authors:** Xinyi Huang, Mayi Zheng, Zetong Zhu, Yuhang Zhang, Kang-Yin Chen

**Affiliations:** 1 Tianjin Key Laboratory of Ionic-Molecular Function of Cardiovascular Disease, Department of Cardiology, Tianjin Institute of Cardiology, Second Hospital of Tianjin Medical University, Tianjin, 300211 China; 2 Graduate College, Tianjin Medical University, Tianjin, 300070 China

**Keywords:** Beta-blockers, Sepsis, Atrial fibrillation, Anti-catecholaminergic therapy, Cardiology, Diseases, Drug discovery, Medical research

## Abstract

Sepsis-induced new-onset atrial fibrillation (NOAF) is a potentially life-threatening arrhythmogenic complication, partially driven by catecholamine-mediated atrial remodeling. While β-adrenergic receptor blockers may mitigate this pathophysiology through sympatholytic and rate-controlling mechanisms, their association with patient survival remains incompletely characterized due to heterogeneous evidence. To elucidate the association between the administration of anti-catecholaminergic antiarrhythmic drugs and all-cause mortality in critically ill patients with sepsis-induced NOAF.This retrospective cohort study utilized data from the Medical Information Mart for Intensive Care-IV database. We included adult patients admitted to a tertiary academic medical center between 2008 and 2019 who fulfilled the Sepsis-3 criteria and developed NOAF during their ICU stays.Based on the initial antiarrhythmic drug administration within 24 h following time-zero, patients were categorized into either β-blocker group or alternative antiarrhythmic drug group. The matched cohort comprised 560 patients (mean age 74.1 [SD 11.5] years; 64.6% male), among whom 280 were assigned to the β-blocker group and 280 to the alternative antiarrhythmic drug group. The β-blocker group demonstrated significantly lower 28-day mortality (36.1 vs. 51.1%; adjusted HR, 0.53; 95% CI, 0.41–0.67; *P* < .001) and 1-year mortality (50.7 vs. 63.6%; adjusted HR, 0.68; 95% CI, 0.55–0.85; *P* = .001). ICU mortality was also reduced in the β-blocker group (22.1 vs. 42.5%; *P* < .001). Furthermore, this group had more vasopressor-free days (median [IQR], 24.0 [18.0–26.0] vs. 16.0 [0.0–23.0]) and ventilator-free days (24.0 [18.0–26.0] vs. 17.0 [0.0–24.0]) (both *P* < .001). Safety outcomes included less bradycardia (12.5 vs. 20.0%; *P* = .016) but more hypotension (26.4 vs. 18.6%; *P* = .039). Subgroup and mediation analyses suggested greater benefit in high-risk patients, partially mediated (15.92%) by reduced vasopressor requirements. In critically ill patients with sepsis-induced NOAF, early β-blocker therapy was associated with markedly lower 28-day and 1-year mortality, along with decreased organ-support burden, warranting prospective trials are warranted to confirm these survival benefits.

**Supplementary Information:**

The online version contains supplementary material available at 10.1038/s41598-026-51249-9.

## Introduction

 Sepsis, which contributes to over 11 million annual deaths globally^1^, predisposes patients to an at least 4-fold increased risk of NOAF compared to non-septic hospitalized individuals^2^. The incidence of sepsis-associated NOAF ranges widely from 1.9% to 43.9%^3^, driven primarily by heterogeneity in illness severity, with the highest rates up to 46% observed among patients with septic shock^4^. Sepsis and NOAF exhibit synergistic detrimental effects, potentially establishing a mutually aggravating cycle that worsens patient prognosis.Beyond prolonging hospital stays^4,5^, NOAF acts as an independent predictor for a spectrum of adverse outcomes, encompassing increased all-cause mortality^2,3,6^, higher AF recurrence^7^, and elevated risks of rehospitalization for heart failure and ischemic stroke^8,9^.The underlying pathophysiological mechanisms are multifactorial and remain incompletely elucidated. Among these, the hyperadrenergic state characteristic of sepsis constitutes a prominent contributor that promotes catecholamine-mediated atrial remodeling. Preclinical studies suggest that this process begins with excessive β-adrenergic stimulation, which increases L-type calcium current and leads to intracellular calcium overload. This cellular disturbanceis thought to promote delayed afterdepolarizations and triggered activity, serving as the initiating focal source for the arrhythmia^10^. At the tissue level, these changes are believed to contribute to a shortening of the atrial effective refractory period and increased heterogeneity in conduction, thereby creating a functional substrate that facilitates re-entry and AF maintenance^11^. If sustained, this pro-fibrillatory environment is further compounded by sympathetic neural hyperinnervation and the activation of pathways leading to interstitial fibrosis, resulting in more permanent structural alterations that stabilize AF and increase its recurrence risk^12,13^. This entire remodeling cascade is potentiated by systemic inflammation and may be influenced by genetic factors, culminating in a vulnerable and persistent arrhythmogenic substrate^14^.

By reducing catecholamine vasopressor requirements^15^ and subsequent β-adrenergic-mediated arrhythmogenicity, vasopressin lowers the incidence of atrial fibrillation in distributive shock^16^; however, this benefit is secondary to its catecholamine-sparing effect rather than to any intrinsic antiarrhythmic property.Under the conventional Vaughan Williams classification, β-blockers are categorized as Class II antiarrhythmics, underscoring their electrophysiological properties of ion channel blockade and heart rate control; however, within the hyperadrenergic milieu of sepsis, β-blockers also confer distinctive “anti-catecholamine” macro-benefits, potentially yielding systemic hemodynamic and metabolic protection beyond mere heart rate reduction, thereby implying an organ-protective foundation and possible survival advantage^17–19^.

Multiple randomized trials, including NCT01231698^20^, J-Land 3 S^21^, and Landi-SEP^22^, have established the efficacy of β-blockers for heart rate control in patients with sepsis and atrial fibrillation.However, their impact on 28-day mortality remains inconsistent, with reported rates of 49.4% vs. 80.5% (*P* < .001), 12% vs. 20% (*P* = .22), and non-significant differences, respectively. Notably, the STRESS-L trial^23^ was terminated early due to a potential signal of harm (37.1% vs. 25.4%, *P* = .08), indicating discordance in the net clinical benefit across high-quality studies. A nationwide retrospective cohort study (*n* = 39,693) found that β-blocker use in patients with sepsis and AF was associated with lower in-hospital mortality compared to calcium channel blockers (RR = 0.92), digoxin (RR = 0.79), and notably, amiodarone (RR = 0.64)^24^. Despite these insights, head-to-head comparative studies of β-blockers versus other commonly used agents like amiodarone, diltiazem, or digoxin specifically for sepsis-related NOAF are currently lacking.

Utilizing the MIMIC-IV database, this study aims to compare the effects of an early β-blocker strategy versus alternative antiarrhythmic drug strategy on short- and long-term mortality, as well as organ support requirements, in patients with sepsis-associated NOAF. Furthermore, we seek to explore whether any potential survival benefit is linked to the mitigation of sympathetic storm-mediated multi-organ injury.

## Methods

## Study design and population

This retrospective cohort study used the MIMIC-IV database (version 3.1), a publicly available, de-identified clinical database of over 450,000 hospital stays for more than 190,000 patients at Beth Israel Deaconess Medical Center (Boston, Massachusetts) between 2008 and 2019. Institutional review boards of MIT and BIDMC approved the use of the database for research, waiving informed consent.

Sepsis was defined per Sepsis-3 criteria^25^ as an acute increase in SOFA score ≥ 2 from baseline with suspected or confirmed infection, evidenced by antibiotic administration or body fluid culture within 48 h before to 24 h after SOFA assessment. Neonatal and puerperal sepsis were excluded.The onset of the first electrocardiographically confirmed NOAF episode was defined as time-zero.

**Fig. 1:**
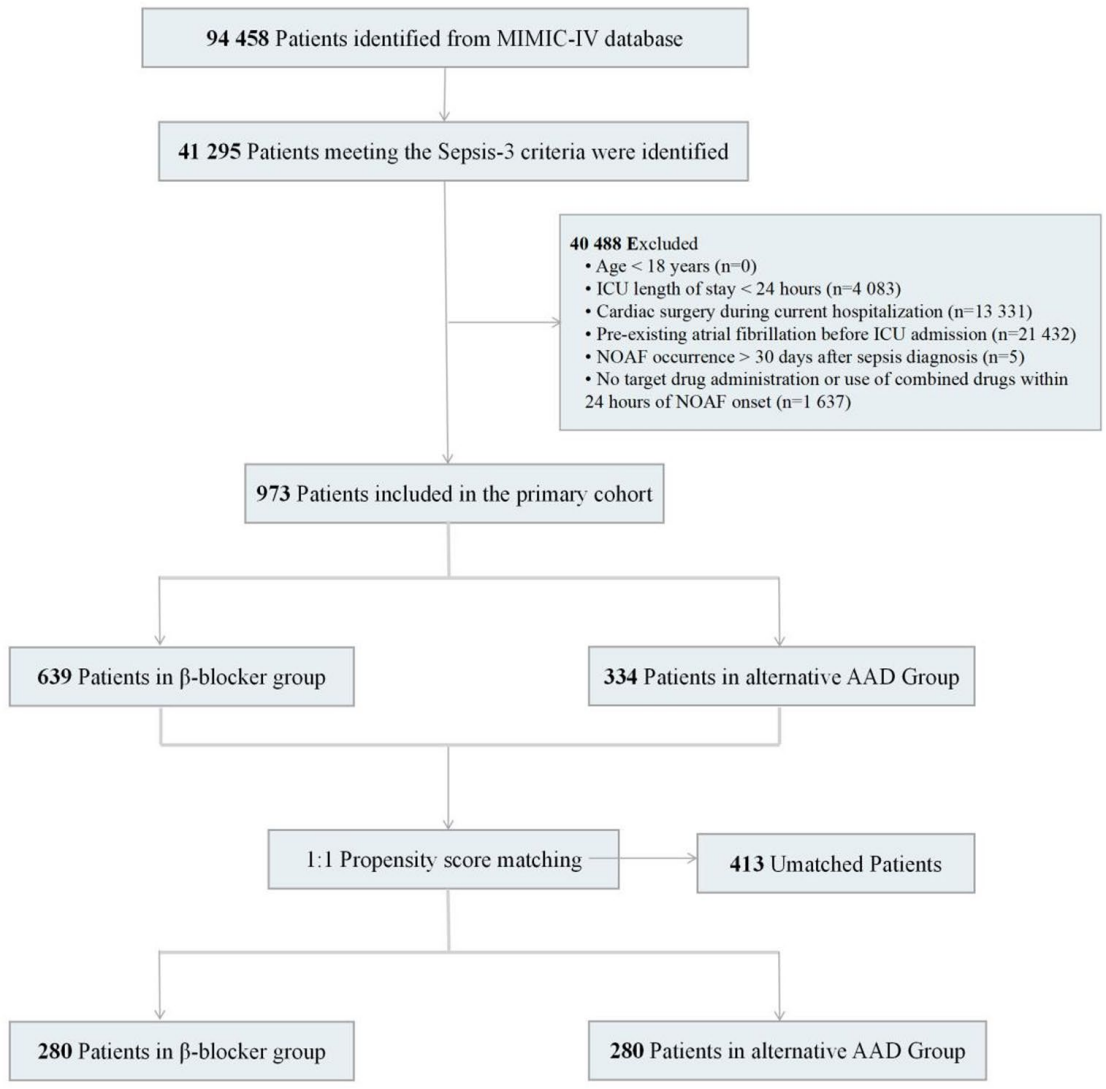
Flowchart of patient enrollment. MIMIC-IV indicates medical information mart in intensive care-IV; ICU, intensive care unit; NOAF, new-onset atrial fibrillation; AAD, antiarrhythmic drug.

From an initial screening of 41,295 patients meeting the Sepsis-3 criteria, the study population was selected based on the following criteria: (1) age ≥ 18 years; (2) ICU length of stay ≥ 24 h; (3) NOAF confirmed by bedside electrocardiographic monitoring, occurring after ICU admission and within 30 days of sepsis diagnosis; (4) administration of either a β-blocker or alternative antiarrhythmic drug antiarrhythmic agent within 24 h of NOAF onset, while excluding patients who received no such pharmacological treatment or received a combination of agents from different groups; (5) no prior history of AF; (6) no cardiac surgery, including valve surgery or coronary artery bypass grafting, during the index hospitalization.; and (7) for patients with multiple ICU admissions, only the first ICU stay was included. The patient selection process is detailed in Fig. 1.

### Exposure and outcomes

Based on standardized medication records within 24 h of NOAF diagnosis, patients were categorized into two groups based on medication administration: the β-blocker group or alternative antiarrhythmic drug group.The β-blocker group comprised patients receiving β-adrenergic antagonists (metoprolol, esmolol, propranolol, atenolol, bisoprolol, labetalol, carvedilol, or sotalol). alternative antiarrhythmic drug group group included those administered other antiarrhythmics (amiodarone, diltiazem, verapamil, digoxin, ibutilide, or propafenone), which act primarily via ion channel blockade or vagal modulation rather than β-receptor inhibition.

The primary outcome was defined as 28-day all-cause mortality. Secondary outcomes comprised 1-year mortality, ICU mortality, successful cardioversion rates at 12/24 hours, and vasopressor-free and ventilator-free days. Safety monitoring focused on episodes of bradycardia and hypotension throughout the predefined exposure window.

### Propensity score matching

To minimize potential confounding, we performed propensity score matching (PSM). A multivariable logistic regression model was constructed using prespecified covariates to estimate each patient’s propensity for receiving β-blocker versus alternative antiarrhythmic drug therapy (eMethods 1). We performed 1:1 propensity score matching using a nearest-neighbor algorithm with a caliper width of 0.1 SD. Covariate balance was evaluated by comparing absolute standardized mean differences (SMD) before and after matching, where SMD > 0.1 was considered indicative of meaningful imbalance.

### Subgroup analyses

In the matched cohort, subgroup analyses for 28-day mortality were performed based on the following variables: age, sex, history of coronary artery disease and heart failure, SOFA score, lactate level, creatinine level, hemoglobin level, heart rate, requirement for invasive mechanical ventilation, and number of vasopressor types.

### Sensitivity analyses

To evaluate the robustness of the primary findings, three sensitivity analyses were performed. First, the association between β-blocker exposure and study outcomes was quantified using a multivariable Cox model in the overall pre-matched cohort. Second, ICU-acquired pressure injury, defined as new-onset pressure ulcers occurring after ICU admission, was captured by structured nursing assessments in the MIMIC-IV database and served as a negative control outcome, given that its etiology is independent of antiarrhythmic drug choice.Finally, the exposure definition was extended to 48 h post-NOAF onset, with the treatment effect re-estimated in a re-matched cohort.

### Statistical analysis

Missing data were handled using multiple imputation with chained equations (MICE), generating 5 imputed datasets under the missing-at-random assumption (eTable 1). Continuous variables were reported as mean (SD) or median (IQR) and compared using t-tests or Wilcoxon rank-sum tests; categorical variables were expressed as frequencies (%) and analyzed with χ²or Fisher’s exact tests. HRs with 95% CIs were derived from Cox proportional hazards models, adjusting for age, sex, vital signs, comorbidities, laboratory values, vasopressor use, and SOFA score. Survival distributions were visualized using Kaplan-Meier curves and compared via log-rank test. To account for competing risks, subdistribution HRs were estimated using Fine-Gray models, and cumulative incidence functions were plotted. All analyses were performed in R v4.5.1, with two-sided *P* < .05 considered significant.

## Results

### Study population and baseline characteristics

We screened eligible patients as detailed in Fig. 1, which yielded a final cohort of 973 patients, comprising 639 assigned to β-blockers and 334 to alternative antiarrhythmic drug. Following 1:1 propensity score matching, 560 patients (280 in each group) were included in the analysis, with balanced baseline characteristics detailed in Table 1.In the overall cohort, patients in the β-blocker group were older, had a higher prevalence of stroke and hypertension, required a higher baseline norepinephrine equivalent dose but fewer concurrent vasoactive agents, and had lower SOFA scores and lower proportions receiving continuous renal replacement therapy and invasive mechanical ventilation. After matching, all covariates were well-balanced, with absolute SMDs below 0.10 (Table 1, eFig. 1). The distribution of propensity scores before and after matching is shown in eFigure 2. In the matched cohort, metoprolol was administered to 90.0% of patients in the β-blocker group (median dose 20 mg/day; maximum 400 mg/day). In the alternative antiarrhythmic drug group, 46.4% received amiodarone (median dose 450 mg/day; maximum 1200 mg/day) and 40.7% received diltiazem (median dose 120 mg/day; maximum 360 mg/day; eTable 2).

**Table 1:** Baseline characteristics before and after matching.

Characteristic	Before matching			After matching		
	β-blocker group (*N* = 639)	alternative AAD group (*N* = 334)	SMD^a^	β-blocker group (*N* = 280)	alternative AAD group (*N* = 280)	SMD
Age, y	76.66(11.97)	73.50(12.23)	0.260	74.55(12.00)	74.23 (12.35)	0.039
Sex, No. (%)	360 (56.34)	176 (52.69)	0.073	137 (48.93)	138 (49.29)	0.030
Smoking history, No. (%)	151 (23.63)	89 (26.65)	0.070	79 (28.21)	72 (25.71)	0.037
Alcohol use, No. (%)	52 ( 8.14)	37 (11.08)	0.100	30 (10.71)	26 ( 9.29)	0.026
Prior CAD^b^, No. (%)	243 (38.03)	118 (35.33)	0.056	100 (35.71)	98 (35.00)	0.044
Prior HF, No. (%)	273 (42.72)	134 (40.12)	0.053	111 (39.64)	115 (41.07)	0.037
Prior cardiac surgery, No. (%)	75 (11.74)	42 (12.57)	0.026	29 (10.36)	36 (12.86)	0.040
Prior hypertension, No. (%)	485 (75.90)	231 (69.16)	0.151	199 (71.07)	201 (71.79)	0.027
Prior T2DM, No. (%)	197 (30.83)	96 (28.74)	0.046	87 (31.07)	79 (28.21)	0.059
Prior stroke, No. (%)	165 (25.82)	46 (13.77)	0.306	40 (14.29)	42 (15.00)	0.028
Prior COPD, No. (%)	156 (24.41)	107 (32.04)	0.170	94 (33.57)	89 (31.79)	0.043
Prior CKD, No. (%)	243 (38.03)	124 (37.13)	0.019	115 (41.07)	103 (36.79)	0.060
Baseline HR, bpm	114.34 (27.56)	118.98 (12.60)	0.216	120.23 (27.14)	118.29 (12.36)	0.079
Baseline MAP, mmHg	91.81 (24.95)	84.84 (26.87)	0.269	88.87 (23.84)	87.72 (27.75)	0.050
Baseline RR, /min	23.93 (8.60)	24.07 (7.91)	0.017	24.14 (8.27)	24.48 (8.22)	0.073
Baseline temperature, °C	37.01 (0.69)	36.90 (0.62)	0.158	36.96 (0.67)	36.94 (0.60)	0.028
CRRT, No. (%)	13 ( 2.03)	21 ( 6.29)	0.214	9 ( 3.21)	11 ( 3.93)	0.042
Mechanical ventilation, No. (%)	297 (46.48)	190 (56.89)	0.209	147 (52.50)	147 (52.50)	0.039
Baseline NED^c^, µg/kg/min	20.36 (100.88)	7.65 (25.39)	0.173	8.03 (32.35)	6.64 (25.35)	0.033
Baseline vasoactive agents used			0.398			0.043
0	475 (74.33)	187 (55.99)		184 (65.71)	183 (65.36)	
1–2	145 (22.69)	101 (30.24)		83 (29.64)	83 (29.64)	
≥3	19 ( 2.98)	46 (13.77)		13 ( 4.65)	14 ( 5.00)	
SOFA score	3.45 (1.83)	4.14 (2.41)	0.321	3.81 (2.00)	3.80 (2.01)	0.017
pH	7.25 (0.44)	7.30 (0.33)	0.121	7.28 (0.35)	7.30 (0.35)	0.043
WBC count^d^, /mL	13.28 (8.94)	14.36 (10.75)	0.109	13.82 (8.02)	14.48 (11.10)	0.058
Hemoglobin, g/dL	10.44 (2.08)	10.41 (1.97)	0.013	10.27 (2.12)	10.41 (2.00)	0.084
Creatinine, mg/dL	1.46 (1.18)	1.60 (1.22)	0.118	1.49 (1.29)	1.50 (1.16)	0.017
Blood urea nitrogen, mg/dL	33.98 (24.89)	35.21 (23.00)	0.051	34.23 (25.51)	34.51 (23.53)	0.029
Glucose, mg/dL	138.14 (55.30)	150.06 (75.94)	0.179	146.95 (69.38)	147.78 (75.61)	0.021
Lactate, mmol/L	2.27 (1.94)	2.84 (2.70)	0.245	2.46 (2.34)	2.47 (2.10)	0.042
Sodium, mmol/L	138.23 (6.03)	137.46 (6.76)	0.121	137.83 (6.35)	137.99 (6.71)	0.026
Potassium, mmol/L	4.17 (1.36)	4.21 (0.78)	0.039	4.18 (0.78)	4.18 (0.76)	0.030
Magnesium, mg/dL	2.05 (0.37)	2.11 (0.36)	0.143	2.10 (0.41)	2.11 (0.36)	0.018
Calcium, mmol/L	1.87 (1.38)	1.86 (1.67)	0.005	1.87 (1.42)	1.92 (1.68)	0.036
Chloride, mmol/L	103.83 (6.99)	103.59 (7.40)	0.033	103.16 (7.21)	103.70 (7.48)	0.043
Bicarbonate, mmol/L	23.03 (5.09)	22.46 (5.24)	0.110	23.00 (5.38)	22.90 (4.91)	0.029

^a^Data are presented as mean (standard deviation) for continuous variables and frequency (percentage) for categorical variables. Standardized mean difference (SMD) was used to assess balance between groups, with SMD < 0.1 generally considered to indicate negligible differences.

^b^CAD, coronary artery disease; HF, heart failure; T2DM, type 2 diabetes mellitus; COPD, chronic obstructive pulmonary disease; CKD, chronic kidney disease; HR, heart rate; MAP, mean arterial pressure; RR, respiratory rate; CRRT, continuous renal replacement therapy; NED, norepinephrine equivalent dose; SOFA, Sequential Organ Failure Assessment; SMD, standardized mean difference.

^c^Norepinephrine equivalent dose was calculated as: Norepinephrine + Epinephrine + (Phenylephrine ÷ 10) + (Dopamine ÷ 100) + (Vasopressin × 2.5 ÷ 60), expressed in mcg/kg/min.

^d^All laboratory values are reported in conventional clinical units. International System of Units (SI) conversion factors: white blood cell count (×10⁹/L) ×1; hemoglobin (g/L) ×10.0; creatinine (µmol/L) ×88.4; blood urea nitrogen (mmol/L) ×0.357; glucose (mmol/L) ×0.0555; magnesium (mmol/L) ×0.4114.

### Association with 28-day mortality

**Fig. 2:**
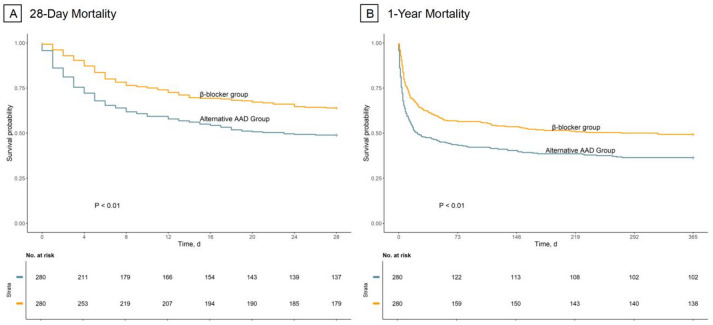
Kaplan-Meier curves for 28-day and 1-year all-cause mortality. Data are from the Medical Information Mart for Intensive Care (MIMIC)-IV database; AAD, antiarrhythmic drug.

In the matched cohort, the 28-day mortality rate was 36.1% (101/280) in the β-blocker group compared to 51.1% (143/280) in alternative antiarrhythmic drug group.The β-blocker group exhibited a significantly lower 28-day mortality rate (HR, 0.53; 95% CI, 0.41–0.67; *P* < .001; Fig. 2). This association remained significant after multivariable adjustment (adjusted HR, 0.68; 95% CI, 0.55–0.85; *P* = .001; Table 2).

**Table 2:** Association of NOAF medication with mid- to long-term mortality in the matched cohort.

Outcomes	β-blocker group	alternativeAAD group	Univariable HR (95% CI)	*P* value	Multivariable HR (95% CI)^a^	*P* Value
28-day mortality	101/280 (36.1%)	143/280 (51.1%)	1.72 (1.29, 2.30)	< 0.001	1.90 (1.12, 3.23)	0.017
1-year mortality	142/280 (50.7%)	178/280 (63.6%)	1.53 (1.21, 1.94)	< 0.001	1.68 (1.13, 2.51)	0.011

AAD, antiarrhythmic drug; HR, hazard ratio; CI, confidence interval.

^a^Multivariable model adjusted for: age, sex, mean arterial pressure, heart rate, body temperature, prior stroke, prior coronary artery disease, prior heart failure, prior chronic obstructive pulmonary disease, prior hypertension, blood pH, lactate, calcium, glucose, magnesium, blood urea nitrogen, number of vasoactive agents, baseline cumulative norepinephrine-equivalent dose, and SOFA score.

### Extended clinical outcomes

In the matched cohort, 1-year mortality was 50.7% (142/280) in the β-blocker group compared with 63.6% (178/280) in the alternative antiarrhythmic drug group. This difference was statistically significant (unadjusted HR, 1.53; 95% CI, 1.21–1.94; *P* < .001; adjusted HR, 1.68; 95% CI, 1.13–2.51; *P* = .011; Table 2; Fig. 2).The median ICU length of stay (LOS) for the 560 matched patients was 5.1 days. ICU mortality was significantly higher in the alternative antiarrhythmic drug group than in the β-blocker group (42.5% vs. 22.1%; adjusted OR, 2.95; 95% CI, 1.89–4.61; *P* < .001; Table 3). Analysis using a competing risk model, with in-ICU death as the event of interest and alive discharge as the competing event, indicated that patients in the alternative antiarrhythmic drug group had a significantly higher risk of in-ICU mortality compared to the β-blocker group (sHR, 1.38; 95% CI[1.13–1.69]; *P* = .002; adjusted sHR, 1.56; 95% CI[1.20–2.04]; *P* = .002; eTable 3).

**Table 3:** Comparison of secondary and safety outcomes by group.

Outcomes	Variables	β-blocker group(*n* = 280)	alternative AAD group(*n* = 280)	OR(95%CI)^a^	*P* value	Adjusted OR (95% CI)^b^	Adjusted *P* value
Secondary outcomes	ICU mortality	62 (22.1%)	119 (42.5%)	2.55 (1.73–3.78)	2.6 × 10⁻⁶	2.95 (1.89–4.61)	2.0 × 10⁻⁶
	12 h conversion success	133 (47.5%)	113 (40.4%)	0.73 (0.52–1.03)	0.074	0.67 (0.45–0.98)	0.040
	24 h conversion success	150 (53.6%)	130 (46.4%)	0.77 (0.54–1.09)	0.135	0.69 (0.47–1.03)	0.069
Safety outcomes	24 h new-onset bradycardia	35 (12.5%)	56 (20.0%)	1.90(1.10–3.27)	0.021	1.75 (1.11–2.78)	0.016
	24 h new-onset hypotension	74 (26.4%)	52 (18.6%)	0.66 (0.43–1.01)	0.055	0.61 (0.39–0.98)	0.039

Data are presented as n (%). Unadjusted and adjusted odds ratios (ORs) with 95% confidence intervals (CIs) are shown. AAD, antiarrhythmic drug; ICU, intensive care unit.

^a^Unadjusted and adjusted odds ratios (OR) with 95% confidence intervals (CI) were derived from logistic regression models.

^b^Multivariable model adjusted for: age, sex, mean arterial pressure, heart rate, body temperature, prior stroke, prior coronary artery disease, prior heart failure, prior chronic obstructive pulmonary disease, prior hypertension, blood pH, lactate, calcium, glucose, magnesium, blood urea nitrogen, number of vasoactive agents, baseline cumulative norepinephrine-equivalent dose, and SOFA score.

Patients in the alternative antiarrhythmic drug group had a significantly higher risk of fewer ventilator-free days (POR, 0.39; 95% CI, 0.28–0.55; *P* < .001) and fewer vasopressor-free days (POR, 0.46; 95% CI, 0.34–0.64; *P* < .001).The proportion of patients requiring continuous vasopressor support was significantly higher in the alternative antiarrhythmic drug group (51.8% vs. 36.1%). Similarly, the proportion requiring continuous mechanical ventilation was significantly higher in the alternative antiarrhythmic drug group (55.7% vs. 36.8%; eTable 4).The 12-hour successful conversion rate was significantly lower in the alternative antiarrhythmic drug group (40.4% vs. 47.5%; OR, 0.67; 95% CI[0.45–0.9]8; *P* = .040). In contrast, the 24-hour conversion rate was not significantly different between the groups (46.4% vs. 53.6%; OR, 0.69; 95% CI[0.47–1.03]; *P* = .069; Table 3).Sensitivity analysis excluding electrical cardioversion cases yielded consistent findings and demonstrated a significantly lower 24-hour conversion rate in the alternative antiarrhythmic drug group.

### Safety analysis

Regarding safety outcomes within the first 24 h after time-zero, the risk of new-onset bradycardia was significantly higher in the alternative antiarrhythmic drug group (20.0% vs. 12.5%; OR, 1.76; 95% CI, 1.11–2.78; *P* = .016). In contrast, the risk of new-onset hypotensive events was significantly higher in the β-blocker group (26.4% vs. 18.6%; OR, 0.62; 95% CI, 0.39–0.98; *P* = .039).Drug-specific dose-response data indicate that the lower bradycardia risk with β-blockers cannot be explained by insufficient dose alone (eTable 5).

### Clinical subgroup investigations

In the overall population, the β-blocker treatment strategy was significantly associated with lower 28-day mortality (HR, 0.58; 95% CI, 0.43–0.78; *P* < .001), corresponding to an absolute risk reduction of 15.7%.Subgroup analyses revealed significant effect heterogeneity, with significant interactions observed between the treatment strategy and age, heart rate, hemoglobin level, and history of coronary heart disease (P for interaction = 0.013, 0.012, 0.023, and 0.002; Fig. 4). The association between β-blocker treatment and improved survival was more pronounced in patients with the following characteristics: age < 75 years, heart rate ≥ 110 beats/min, hemoglobin level > 10.0 g/dL, and a history of coronary heart disease.

**Fig. 3:**
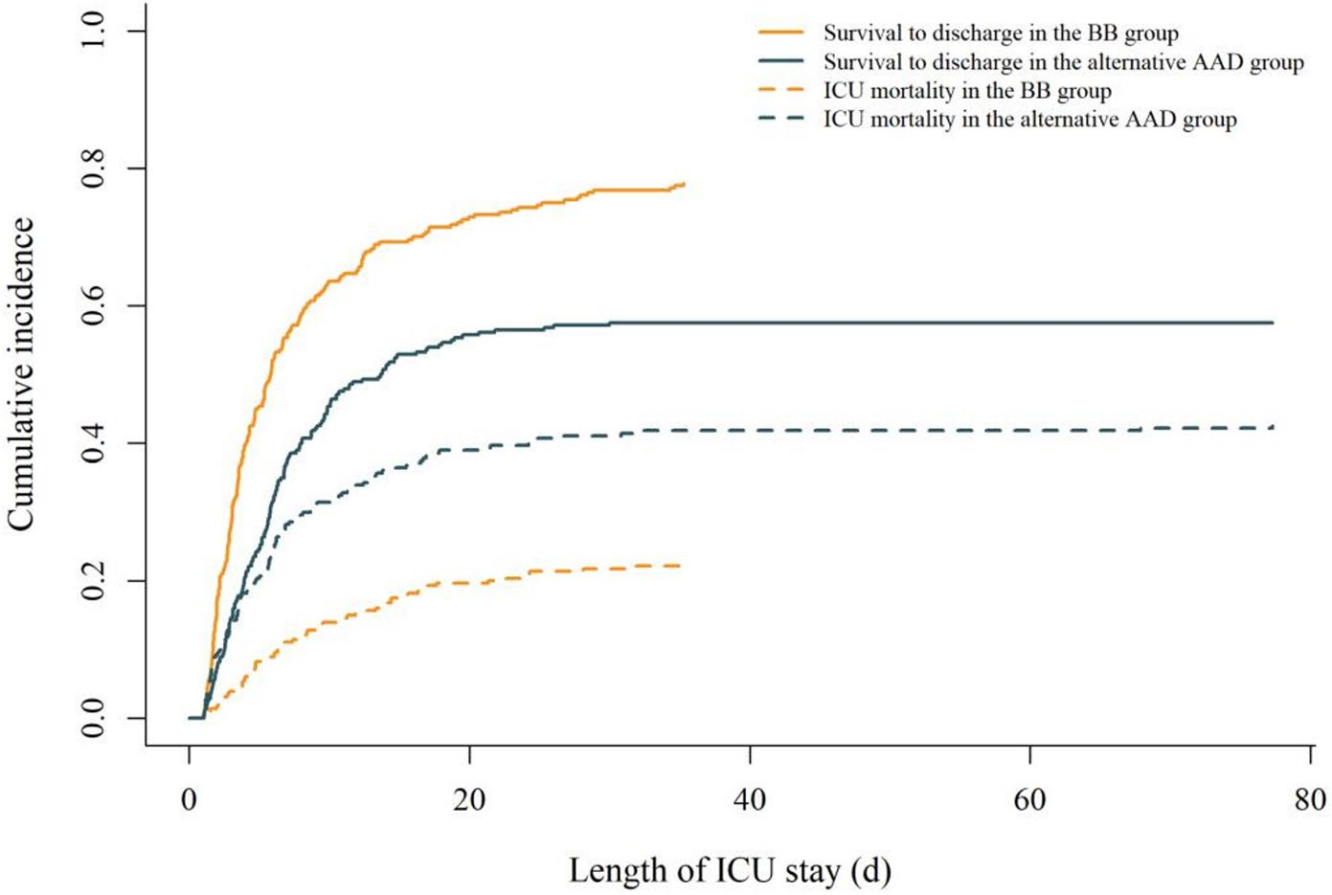
Cumulative incidence curves by treatment group based on the fine-gray competing risk mode. Cumulative incidence of in-ICU death (dashed lines) and alive ICU discharge (solid lines) over ICU length of stay, comparing BB therapy (blue) with other antiarrhythmic agents (red).BB, β-blocker; AAD, antiarrhythmic drug.

**Fig. 4:**
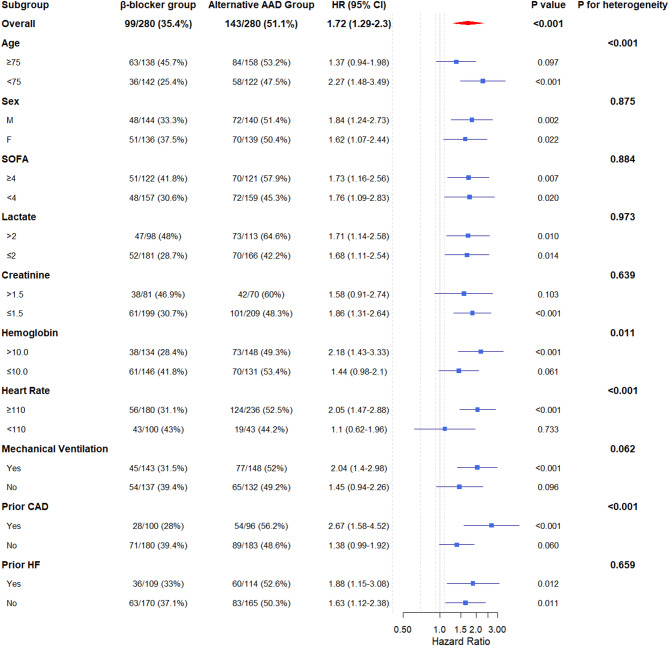
Subgroup analysis of 28-day mortality by study group in the matched cohort. Data are presented as event rates (n/N, %), HR (95% CI), and interaction P value for each subgroup. CAD, coronary artery disease; HF, heart failure; SOFA, Sequential Organ Failure Assessment.BB,β-blocker; ICU, intensive Care Unit; AAD, antiarrhythmic drug; HR, hazard ratio; CI, confidence interval; SOFA, Sequential Organ Failure Assessment; CAD, coronary artery disease; HF, heart failure.

### Comparative efficacy and dose-response analyses

Given heterogeneity within drug groups, we compared the three most frequently used agents: metoprolol (*n* = 252), amiodarone (*n* = 130), and diltiazem (*n* = 114), representing 496 patients (88.6% of matched cohort). After multivariable adjustment, amiodarone was associated with significantly higher 28-day mortality than metoprolol (aHR, 1.70; 95% CI, 1.23–2.34; *P* = .002;eTable 6), whereas diltiazem showed a trend toward increased risk (aHR, 1.40; 95% CI, 0.99–1.98; *P* = .057). Similar patterns were observed for 1-year mortality. Interaction tests did not support significant effect modification by baseline vasopressor use (amiodarone: *P* = .320; diltiazem: *P* = .180), suggesting the metoprolol survival advantage is unlikely to be fully explained by confounding by indication related to differential vasopressor requirements.

We performed an exploratory dose-stratified analysis in patients treated with metoprolol (*n* = 252). After multivariable adjustment and using the low-dose group (< 10 mg/24 h) as reference, the medium-dose group (10–40 mg/24 h) showed a comparable mortality risk (aHR 0.80, 95%CI 0.51–1.28; *P* = .358), while the high-dose group (> 40 mg/24 h) demonstrated a significantly lower risk (aHR 0.38, 95%CI 0.19–0.76; *P* = .006;eTable 7). A significant graded dose–response relationship was observed across the three dose groups (P for trend = 0.007). The pattern was consistent for 1-year mortality (P for trend = 0.004).

### Sensitivity analyses

Use of alternative antiarrhythmic drug was associated with significantly higher 28-day mortality in the original cohort (unadjusted HR, 1.87; 95% CI, 1.54–2.28; *P* < .001), with crude rates of 54.2% (181/334) versus 36.0% (230/639) in the β-blocker group. This association persisted after multivariable adjustment (aHR, 1.78; 95% CI, 1.24–2.55; *P* = .002; eTable 8) and was consistent for 1-year mortality (eFigure 3).Extending the exposure window to 48 h yielded results consistent with the primary analysis: the mortality benefit with β-blockers persisted, while the early conversion advantage was attenuated and the between-group difference in hypotension risk narrowed (eTable 9).To address unmeasured confounding, we analyzed ICU-acquired pressure injury as a negative control outcome—a nonspecific marker of general illness severity that is not plausibly directly affected by choice of atrial fibrillation treatment. No significant difference was found between the alternative antiarrhythmic drug (27.9%) and β-blocker (29.3%) groups (OR, 1.007; 95% CI, 0.689–1.471; *P* = .971), providing a benchmark suggesting that the groups were balanced on underlying illness severity, thereby supporting the internal validity of the primary mortality comparison.

### Analysis of medication timing in relation to rate and rhythm control

The success of rhythm control in sepsis-associated NOAF demonstrated a time-sensitive nature. Each one-hour delay in antiarrhythmic administration was associated with a significant reduction in the probability of sinus rhythm restoration across both treatment strategies (adjusted HRs ~ 0.975; *P* < .001; eTable 10).In contrast, treatment timing showed no significant association with the rate of heart rate reduction in either the β-blocker (β= -0.015, 95% CI: -0.162 to 0.132; *P* = .841) or alternative antiarrhythmic drug group (β= -0.026, 95% CI: -0.182 to 0.130; *P* = .747; eTable 11). Furthermore, no between-group differences were observed in rhythm or rate control efficacy (*P* > .05 for all comparisons), and these null findings remained consistent in sensitivity analyses employing multiple imputation and bootstrap validation.

### Mediation role of ΔNED

We performed mediation analysis to assess whether the reduction in exogenous vasopressor requirements (ΔNED) mediated the association between β-blocker use and lower 28-day mortality, with the alternative antiarrhythmic drug strategy as the reference.Mediation analysis revealed that ΔNED explained 15.92% of the total protective effect on 28-day mortality (indirect effect: 0.097; 95% CI, 0.029–0.164; *P* = .005). At 1 year, the proportion mediated increased to 17.10% (indirect effect: 0.085; 95% CI, 0.019–0.152; *P* = .013). The direct effects remained significant at both timepoints (*P* = .001; eFigure 4, eTable 12).

## Discussion

In this study, early β-blocker use was associated with reduced mortality in sepsis-associated NOAF compared to other antiarrhythmic agents, a benefit that persisted across sensitivity analyses and patient subgroups.Furthermore, earlier pharmacologic intervention was linked to enhanced rhythm control rather than heart rate control. While rhythm and rate control efficacy were comparable between strategies, the β-blocker approach offered a distinct benefit through an enhanced safety profile and reduced need for advanced organ support.Mechanistically, mediation analysis indicated that approximately 16% of the survival benefit associated with β-blocker could be explained by reduced catecholamine-driven vasopressor dependency.This modest proportion is both methodologically expected and physiologically revealing. The mediator quantifies the change in exogenous catecholamine support. In sepsis, however, a profound endogenous catecholamine surge occurs early and may precede any requirement for exogenous vasopressor support^26,27^. Supporting this possibility, the survival benefit afforded by metoprolol remained substantial even among the 64% of patients who never required vasopressors at enrolment (P for interaction > 0.3). Roughly 84% of the survival benefit remained unmediated; this residual effect is consistent with partial mitigation of the endogenous catecholaminergic surge, although its exact contribution remains undetermined. The conservative starting doses observed in our cohort reflect real-world ICU practice, where clinicians prudently initiate therapy with low doses to assess tolerability before considering titration. Importantly, despite this cautious approach, metoprolol demonstrated clear pharmacodynamic activity, achieving rate control comparable to diltiazem with significantly less bradycardia. Furthermore, the significant dose-response gradient provides strong biological plausibility for a causal relationship and suggests that optimized, individually titrated regimens may yield even greater therapeutic benefits than those observed with the initial conservative dosing in our study. We emphasize that our dose-stratified analysis was exploratory and hypothesis-generating, and the cutoffs used should not be interpreted as clinical dosing recommendations.Collectively, these findings suggest that the therapeutic role of β-blockers in sepsis-associated NOAF extends beyond conventional rate control. We propose that beta-blockers may function as disease-modifying agents targeting fundamental sepsis pathophysiology, with their anti-catecholamine properties serving as the foundation for observed clinical benefits.

Long-term follow-up studies indicate that the recurrence rates of atrial fibrillation in sepsis-associated NOAF patients reach 44% at 1 year and 55% at 5 years^8^, suggesting that this condition is not merely a transient, self-limiting arrhythmia of the acute phase.Early β-blocker therapy demonstrated a survival benefit in septic patients with NOAF, which was particularly marked in those with heightened sympathetic tone (heart rate ≥ 110 bpm) and a high-risk profile encompassing age < 75 years, hemoglobin > 10 g/dL, and history of coronary artery disease.Our primary results align with previous reports by Walkey^23^ and Oprea^28^ et al. describing survival advantages with beta-blocker therapy. The divergent results between our study and the CAFÉ trial^29^, considered alongside insights from a recent synthesis of the evidence^30^, underscore that heterogeneity in patient populations and critical variations in intervention timing are central to understanding the efficacy of β-blockers in sepsis. While clinical practice has often favored initiating these agents in hemodynamically stable patients, efficacy among appropriately resuscitated patients appears determined more by precise physiological phenotypes than by stability alone, as evidenced by the benefit of propafenone in septic shock patients with a non‑dilated left atrium^31^. Equally critical is the narrow therapeutic window, where premature administration may risk hemodynamic instability and excessive delay may miss the optimal period for modulating sustained sympathetic drive^30,32^. These collective differences underscore the complexity of sepsis-associated NOAF as a distinct clinical entity, thus warranting prospective studies to identify and validate these responsive sub-phenotypes. Our subgroup analyses identify high sympathetic tone as a potential biomarker for treatment response, supporting a more targeted application strategy.

When selecting a rate-control agent in this setting, the risk of bradycardia is paramount. We observed a lower incidence of bradycardia with metoprolol than with amiodarone or diltiazem.Consequently, the lower bradycardia risk reflects a more favourable safety profile of β-blockade at this effective yet cautious dose—a strategy aligned with safety-oriented practice in sepsis^33–35^. In contrast, the higher bradycardic liability of amiodarone and diltiazem stems from their direct sino-atrial node suppression, as documented in post-operative and thoracic ICU cohorts^36,37^. Taken together, metoprolol offers an advantageous efficacy-safety balance in early sepsis-related NOAF, achieved through a rational starting dose, demonstrable rate control, and a mechanistic profile distinct from multi-channel or calcium-channel blockers.

Our findings are consistent with the hypothesis that the survival benefit of beta-blockers may derives from their specific antagonism of sepsis-associated catecholamine excess, fundamentally distinguishing them from the multi-channel blockade of amiodarone or calcium channel blockade of diltiazem. This mechanistic difference may yield several pleiotropic effects. Through modulation of the β2AR-Gi pathway, beta-blockers may attenuate catecholamine-mediated calcium overload, oxidative stress, and apoptosis, thereby reducing direct myocardial toxicity and providing cardioprotection^38^.Additionally, by antagonizing the β2-AR-cAMP/PKA axis, β-blockers might suppress CTGF/VEGF paracrine signaling between cardiomyocytes and immune cells, potentially mitigating systemic inflammation and organ dysfunction^39^. Transcriptomic profiling reveals approximately 43% repression of mitochondrial oxidative phosphorylation and TCA-cycle genes in human septic myocardium, a bioenergetic deficit that β-blockade may rectify by reducing heart rate and myocardial oxygen demand^35,40^.

We further propose the concept of “physiologically coordinated rate control” to describe the heart rate reduction achieved with beta-blockers. This represents an adaptive response synchronized with the overall pathophysiological state, contrasting with the forced sinus node suppression characteristic of alternative rate-control agents. The latter approach, while effectively reducing heart rate, may compromise preserved compensatory mechanisms in sepsis, potentially leading to inadequate cardiac output and impaired tissue perfusion. This may explain the higher incidence of bradycardia and increased organ support requirements observed in the alternative antiarrhythmic drug group. In hemodynamically stable patients with severe sepsis, beta-blocker–mediated rate control appears safe and well-tolerated, with preservation of blood pressure, stroke volume, lactate clearance, and central venous oxygen saturation despite reduced cardiac output^41^. Notably, each one-hour delay in treatment initiation was associated with an approximately 2.3% reduction in the probability of sinus rhythm restoration, without affecting the rate of heart rate reduction. This temporal pattern suggests that the primary benefit of early intervention lies in electrophysiological stabilization rather than mere ventricular rate control.

Several limitations warrant consideration. First, the observational design precludes definitive causal inferences, and residual confounding from factors such as physician prescribing preferences or unmeasured hemodynamic variables cannot be completely excluded. Although we employed propensity score matching, multivariable adjustment, and negative control analyses to address this concern, residual bias may persist. Second, as our data originated from the MIMIC database and our cohort included patients across the spectrum of sepsis severity—with only a subset meeting criteria for septic shock—external validation across diverse clinical settings is necessary to establish generalizability, and our findings should be extrapolated to the most hemodynamically unstable patients with septic shock with caution.Third, this classification scheme may not fully capture the complex and overlapping pharmacology of these drugs, encompassing both the shared propanolamine-derived structure that confers overlapping properties and the data granularity limitations that prevented distinguishing short-acting from long-acting formulations, thus underscoring the challenge of disentangling discrete drug effects in observational analyses. Furthermore, the absence of both detailed echocardiographic, hemodynamic data and serial inflammatory biomarkers further limited our mechanistic exploration of specific phenotypes and pathways. Ultimately, the optimal agent, dosing, timing, and continuation strategy for beta-blockers in this setting should be evaluated in prospective randomized trials.

## Conclusions

This study demonstrates that the therapeutic benefit of early β-blockers in sepsis-associated NOAF transcends simple rhythm management. Its effect is mechanistically linked to mitigating catecholamine toxicity, translating into multimodal survival and organ-sparing advantages. Prospective studies are warranted to determine the optimal agent, dosing, and timing for this therapeutic approach.

## Supplementary Information

Below is the link to the electronic supplementary material.

Supplementary Material 1

## Data Availability

The MIMIC-IV (v3.1) database is publicly available at https://physionet.org/content/mimiciv/3.1/ under a PhysioNet Credentialed Health Data Use Agreement (DUA).
