# Supplementary material for: Association of anti-catecholaminergic antiarrhythmic drugs with survival in sepsis-associated new-onset atrial fibrillation
Source: Sci Rep. 2026 Jul 6;16:20588. doi: 10.1038/s41598-026-51249-9 (PMC13334026; doi:10.1038/s41598-026-51249-9)
Supplement: Supplementary file 1 — Supplementary Material 1 [file 41598_2026_51249_MOESM1_ESM.docx]

**Supplementary Material**

eTable 1. Percentage of Missing Data in the Cohort

eTable 2. Details of Drug Administration

eTable 3. Fine-Gray Competing Risk Models for In-ICU Mortality and Survival to Discharge

eTable 4. Distribution of Vasopressor-Free Days and Ventilator-Free Days and Results of the Proportional Odds Model Analysis

eTable 5. Association of Antiarrhythmic Drug Dose and Early Heart-Rate Reduction with Bradycardia Risk

eTable 6. Baseline Characteristics and Adjusted Mortality Outcomes of Patients Receiving Metoprolol, Amiodarone, or Diltiazem

eTable 7. Dose-Response Relationship of Metoprolol and Mortality

eTable 8. Cox Proportional Hazards Models for Treatment Strategies in the Original Cohort

eTable 9. Association Analysis of Primary and Secondary Outcomes in the Matched Cohort with Extended Dosing Window to 48 Hours

eTable 10. Association Between Timing of Early Medication and Speed of Sinus Rhythm Restoration

eTable 11. Association Between Timing of Early Medication and Rate of Heart Rate Reduction

eTable 12. Mediation Analysis of the Effect of β-blockers on Mid- to Long-Term Mortality via ΔNED

eFigure 1. Density Distribution of Propensity Scores

eFigure 2. Covariate Balance Plot

eFigure 3. Kaplan-Meier Curves for 28-Day and 1-Year Mortality by Treatment Strategy in the Original Cohort

eFigure 4. Mediation Pathway Diagram and Bootstrap Mediation Effect Distribution

eTable 1. Percentage of Missing Data in the Cohort

| **Variables** | **N=937** |
| --- | --- |
| Lactate | 18.4% |
| pH | 5.9% |
| WBC | 5.0% |
| Magnesium | 2.5% |
| Creatinine | 1.8% |
| BUN | 1.8% |
| Glucose | 1.7% |
| Hemoglobin | 1.6% |
| Temperature | 0.2% |
| Potassium | 0.2% |
| Sodium | 0.2% |
| Calcium | 0.2% |
| Chloride | 0.2% |
| Bicarbonate | 0.2% |

WBC, white blood cell count; BUN, blood urea nitrogen.

eTable 2. Details of Drug Administration

| **Type** | **Unmatched** | **Matched** |
| --- | --- | --- |
| β-blockers | N = 639 | N = 280 |
| Metoprolol | 557（87.17%） | 252（90.0%） |
| Atenolol | 11（1.72%） | 1（0.40%） |
| Propranolol | 2（0.31%） | 2（0.71%） |
| Esmolol | 12（1.88%） | 7（2.50%） |
| Carvedilol | 8（1.25%） | 6（2.14%） |
| Labetalol | 46（7.20%） | 11（3.93%） |
| Bisoprolol | 0（0.00%） | 0（0.00%） |
| Sotalol | 3（0.47%） | 1（0.40%） |
| alternative antiarrhythmic drug | N = 334 | N = 280 |
| Amiodarone | 169（50.60%） | 130（46.43%） |
| Diltiazem | 121（36.23%） | 114（40.71%） |
| Verapamil | 1（0.30%） | 1（0.40%） |
| Digoxin | 42（12.57%） | 34（12.14%） |
| Ibutilide | 1（0.30%） | 1（0.40%） |

| Propafenone | 0（0.00%） | 0（0.00%） |
| --- | --- | --- |

eTable 3. Fine-Gray Competing Risk Models for In-ICU Mortality

| Model type | sHR^a^ | 95% CI | P value | Coefficient | SE |
| --- | --- | --- | --- | --- | --- |
| Univariate | 1.3799 | 1.1274-1.6889 | 0.0018 | 0.322 | 0.1031 |
| Multivariate^b^ | 1.5649 | 1.2031-2.036 | 0.0020 | 0.448 | 0.1342 |

sHR, subdistribution hazard ratio;CI, confidence interval;SE, standard error.

^a^Event of interest: in-ICU death; competing event: alive hospital discharge.

^b^Multivariable model adjusted for: age, sex, mean arterial pressure, heart rate, body temperature, prior stroke, prior coronary artery disease, prior heart failure, prior chronic obstructive pulmonary disease, prior hypertension, blood pH, lactate, calcium, glucose, magnesium, blood urea nitrogen, number of vasoactive agents, baseline cumulative norepinephrine-equivalent dose, and SOFA score.

eTable 4. Distribution of Vasopressor-Free Days and Ventilator-Free Days and Results of the Proportional Odds Model Analysis

| Variable | Group | 0 d, n (%) | 1-27 d, n (%) | 28 d, n (%) | POR (95% CI) | P value |
| --- | --- | --- | --- | --- | --- | --- |
| Vasopressor-Free Days | β-blocker group | 101 (36.1) | 34 (12.1) | 145 (51.8) | 0.46 (0.34-0.64) | <0.001 |
|  | Alternative AAD Group | 145 (51.8) | 49 (17.5) | 86 (30.7) |  |  |
| Ventilator-Free Days | β-blocker group | 103 (36.8) | 135 (48.2) | 42 (15.0) | 0.39 (0.28-0.55) | <0.001 |
|  | Alternative AAD Group | 156 (55.7) | 118 (42.1) | 6 (2.1) |  |  |

POR, proportional odds ratio; AAD, antiarrhythmic drug; d, days.

eTable 5. Association of Antiarrhythmic Drug Dose and Early Heart-Rate Reduction with Bradycardia Risk

| Subgroup | n | Dose, mg/24 h Median [IQR] | ΔHR at 1 h [IQR], bpm | ΔHR at 6 h Median [IQR], bpm | ΔHR at 24 h Median [IQR], bpm | Bradycardia^†^ %[95%CI] |
| --- | --- | --- | --- | --- | --- | --- |
| Metoprolol | 252 | 20 [7.5–40] | −8 [−26.5 to 0.0] | −16 [−39.0 to −1.5] | −24 [−48.0 to −6.0] | 15.51 [11.3 to 20.8] |
| Amiodarone | 130 | 450 [375–600] | −10.5 [-27 to 1.0] | −19.5 [−39.8 to −5.0] | −30.0 [−54.8 to −16.5] | 25.8[18.7 to 34.2] |
| Diltiazem | 114 | 120 [18.8–125] | −12.5 [−25.8 to 2.0] | −18 [−30.8 to −1.0] | −21[−35.5 to −6.2] | 14.8[9.0 to 23.3] |

IQR, interquartile range; ΔHR, change in heart rate from pre-drug baseline; bpm, beats per minute;95%CI,95% confidence interval.

eTable 6. Baseline Characteristics and Adjusted Mortality Outcomes of Patients Receiving Metoprolol, Amiodarone, or Diltiazem

| Characteristic / Outcome | Metoprolol (n=252) | Amiodarone (n=130) | Diltiazem (n=114) | P value | SMD/HR[95CI]^a^ |
| --- | --- | --- | --- | --- | --- |
| Baseline Characteristics | | | | | |
| Age, y | 69.57(11.51) | 58.13(3.02) | 73.76(11.79) | <0.001 | 1.842 |
| Male sex, No. (%) | 127(50.40) | 72(55.38) | 51(44.74) | 0.252 | 0.220 |
| SOFA score | 3.87(2.10) | 4.02(2.23) | 3.58(1.95) | 0.083 | 0.290 |
| Baseline HR, bpm | 122.53(24.98) | 118.48(12.46) | 118.66(13.60) | 0.301 | 0.191 |
| Baseline MAP, mmHg | 87.19(20.02) | 83.71(26.05) | 85.49(22.14) | 0.964 | 0.163 |
| Baseline NED, μg/kg/min, | 7.38(31.32) | 6.91(30.44) | 7.41(30.55) | 0.285 | 0.184 |
| Baseline vasoactive agents used | | | | 0.838 | 0.264 |
| 0 | 161(63.89） | 80(61.53) | 78(68.42) |  |  |
| 1–2 | 78(30.95) | 42(32.30) | 30(26.32) |  |  |
| ≥3 | 13(5.16) | 8(6.15) | 6(5.26) |  |  |
| CRRT, No. (%) | 9(3.57) | 10(7.69) | 2(1.75) | 0.062 | 0.125 |
| Mechanical ventilation, No. (%) | 128(50.79) | 73(56.15) | 60(52.63) | 0.610 | 0.211 |
| Clinical Outcomes | | | | | |
| 28-day mortality, No. (%) | 91(36.11) | 70(53.85) | 51(44.74) | 0.002b | 1.70 [1.23-2.34] |
|  |  |  |  | 0.057c | 1.40 [0.99-1.98] |
| 1-year mortality, No. (%) | 129(51.19) | 84(64.62) | 68(59.65) | 0.006 | 1.52 [1.14-2.02] |
|  |  |  |  | 0.043 | 1.35 [1.00-1.82] |

SMD,standardized mean difference; HR,hazard ratio; CI,confidence interval.

^a^ Cox proportional hazards models were adjusted for age, sex, SOFA score, heart rate, mean arterial pressure, use of vasoactive agents, CRRT, mechanical ventilation, and cumulative norepinephrine dose. Robust standard errors were clustered by matched pairs. Metoprolol served as the reference group. Overall model test P-values were <0.001 for both 28-day and 1-year mortality. No significant treatment-by-vasoactive agent interaction was observed (amiodarone: P=0.320; diltiazem: P=0.180).

ᵇ P value, HR, and 95% CI for amiodarone versus metoprolol.

ᶜ P value, HR, and 95% CI for diltiazem versus metoprolol.

eTable 7. Dose-Response Relationship of Metoprolol and Mortality^a^

| Dose group^b^ | n | 28-day mortality, No. (%) | 1-year mortality, No. (%) | aHR[95% CI]^c^ | | P value | |
| --- | --- | --- | --- | --- | --- | --- | --- |
|  |  |  |  | 28-day | 1-year | 28-day | 1-year |
| Low dose (<10 mg/24h) | 72 | 33 (45.8) | 43 (59.7) | 1.00 (Reference) | 1.00 (Reference) | - | - |
| Medium dose (10-40 mg/24h) | 117 | 44 (37.6) | 62 (53.0) | 0.80 (0.51-1.28) | 0.78 (0.52-1.17) | 0.358 | 0.230 |
| High dose (>40 mg/24h) | 63 | 12 (19.0) | 23 (36.5) | 0.38 (0.19-0.76) | 0.46 (0.27-0.78) | 0.006 | 0.004 |
| P for trend | - | - | - | - | - | 0.007 | 0.004 |

^a^Dose categories were chosen based on the distribution of administered doses in our cohort for exploratory analysis and are not intended as clinical dosing recommendations. This analysis is hypothesis-generating and requires validation in prospective studies.

^b^The dose refers to the total intravenous metoprolol administered in the 24 hours following the onset of NOAF (time-zero).

^c^HRs were adjusted for age, sex, SOFA score, heart rate, mean arterial pressure, use of vasoactive agents, CRRT, mechanical ventilation, and cumulative norepinephrine dose.

eTable 8. Cox Proportional Hazards Models for Treatment Strategies in the Original Cohort

| Outcomes | β-blocker group | Alternative AAD Group | Univariable HR (95% CI) | P value | Multivariable HR (95% CI)^a^ | P value |
| --- | --- | --- | --- | --- | --- | --- |
| 28-day mortality | 230/639 (36.0%) | 181/334 (54.2%) | 1.87 (1.54-2.28) | <0.001 | 1.78 (1.24-2.55) | 0.002 |
| 1-year mortality | 333/639 (52.1%) | 220/334 (65.9%) | 1.62 (1.36-1.92) | <0.001 | 1.58 (1.19-2.10) | 0.001 |

AAD, antiarrhythmic drug; HR, hazard ratio; CI, confidence interval.

^a^The multivariable model was adjusted for covariates identical to those in the primary analysis.

eTable 9. Association Analysis of Primary and Secondary Outcomes in the Matched Cohort with Extended Dosing Window to 48 Hours

| Outcome | Variable | β-blocker group^a^ | Alternative AAD Group | HR^b^/OR^c^ (95% CI) | P value | Adjusted HR/OR (95% CI)^d^ | Adjusted P value |
| --- | --- | --- | --- | --- | --- | --- | --- |
| Primary Outcome | 28-day mortality | 181/429 (42.2%) | 161/267 (60.3%) | 1.71 (1.37, 2.13) | <0.001 | 1.78 (1.20, 2.63) | 0.004 |
| Secondary Outcomes | 1-year mortality | 257/429 (59.9%) | 200/267 (74.9%) | 1.54 (1.28, 1.87) | <0.001 | 1.59 (1.17, 2.16) | 0.003 |
|  | ICU mortality | 108/429 (25.2%) | 120/267 (44.9%) | 2.36 (1.69-3.29) | <0.0001 | 2.41 (1.69-3.43) | <0.01 |
|  | 12h conversion success | 212/429 (49.4%) | 133/267 (49.8%) | 1.05 (0.77-1.43) | 0.770 | 0.95 (0.68-1.33) | 0.781 |
|  | 24h conversion success | 240/429 (55.9%) | 152/267 (56.9%) | 1.08 (0.79-1.47) | 0.636 | 0.98 (0.69-1.38) | 0.895 |
|  | 48h conversion success | 264/429 (61.5%) | 167/267 (62.5%) | 1.09 (0.79-1.50) | 0.604 | 0.99 (0.70-1.41) | 0.960 |
| Safety Outcomes | 24h new-onset bradycardia | 68/429 (15.9%) | 64/267 (24.0%) | 1.73 (1.14-2.63) | 0.010 | 1.68 (1.15-2.46) | 0.008 |
|  | 24h new-onset hypotension | 119/429 (27.7%) | 89/267 (33.3%) | 1.30 (0.93-1.82) | 0.129 | 1.31 (0.93-1.86) | 0.125 |

AAD, antiarrhythmic drug; HR, hazard ratio; OR, odds ratio; CI, confidence interval.

^a^The matched cohort was created using 2:1 PSM with a caliper width of 0.1.

^b^HRs are reported for time-to-event outcomes.

^c^ORs are reported for binary outcomes.

^d^Adjusted models used the propensity score derived from primary analysis covariates.

eTable 10. Association Between Timing of Early Medication and Speed of Sinus Rhythm Restoration

| Group | Model | HR/ΔHR | SE | 95%CI | P value |
| --- | --- | --- | --- | --- | --- |
| β-blocker group | Crude model^a^ | 0.967 | 0.022 | [0.690, 1.244] | 0.014 |
|  | Multivariable model^b^ | 0.977 | 0.019 | [0.926, 1.029] | <0.001 |
| Alternative AAD Group | Crude model | 0.962 | 0.016 | [0.930, 0.995] | <0.001 |
|  | Multivariable model | 0.973 | 0.017 | [0.940, 1.007] | <0.001 |
| β-blocker group vs Alternative AAD Group | Crude model | 0.004 | 0.020 | [-0.062, 0.071] | 0.846 |
|  | Multivariable model | 0.004 | 0.012 | [-0.037, 0.045] | 0.774 |
|  | Model with interaction term^c^ | 0.991 | 8.367 | [-15.407, 17.389] | 0.906 |
|  | Bootstrap analysis^d^ | 0.006 | 0.013 | [-0.037, 0.048] | 0.688 |

AAD, antiarrhythmic drug; HR, hazard ratio (per unit time); ΔHR, difference in hazard ratios; SE, standard error; CI, confidence interval.

^a^The crude model assessed the univariable association.

^b^The multivariable model was adjusted for age, sex, mean arterial pressure, heart rate, body temperature, number of vasoactive agents at baseline, SOFA score, cumulative norepinephrine-equivalent dose in the 24 hours before NOAF, baseline pH, lactate, glucose, blood urea nitrogen, calcium, and magnesium, as well as history of hypertension, heart failure, stroke, coronary heart disease, and COPD.

^c^The model with interaction term tested for effect modification by group.

^d^The bootstrap analysis was performed with 1 000 replications to validate the stability of the ΔHR estimate.

eTable 11. Association Between Timing of Early Medication and Rate of Heart Rate Reduction

| Group | Model | HR/ΔHR | SE | 95%CI | P value |
| --- | --- | --- | --- | --- | --- |
| β-blocker group | Crude model^a^ | 0.099 | 0.098 | [-0.103, 0.301] | 0.322 |
|  | Multivariable model^b^ | -0.015 | 0.073 | [-0.162, 0.132] | 0.841 |
| Alternative AAD Group | Crude model | 0.022 | 0.078 | [-0.131, 0.176] | 0.774 |
|  | Multivariable model | -0.026 | 0.080 | [-0.182, 0.130] | 0.747 |
| β-blocker group vs Alternative AAD Group | Crude model | 0.077 | 0.033 | [-0.042, 0.195] | 0.121 |
|  | Multivariable model | 0.011 | 0.031 | [-0.090, 0.112] | 0.748 |
|  | Model with interaction term^c^ | -0.029 | 0.100 | [-0.225, 0.166] | 0.770 |
|  | Bootstrap analysis^d^ | 0.009 | 0.030 | [-0.089, 0.107] | 0.777 |

AAD, antiarrhythmic drug; HR, hazard ratio (per unit time); ΔHR, difference in hazard ratios; SE, standard error; CI, confidence interval.

^a^The crude model assessed the univariable association.

^b^The multivariable model was adjusted for covariates identical to those used in the primary analysis of eTable 7.

^c^The model with interaction term tested for effect modification by group.

^d^The bootstrap analysis was performed with 1 000 replications to validate the stability of the ΔHR estimate.

eTable 12. Mediation Analysis of the Effect of β-blockers on Mid- to Long-Term Mortality via ΔNED

| Outcome | Parameter | Pooled Estimate^a^ | SE | 95% CI LL | 95% CI UL | P value |
| --- | --- | --- | --- | --- | --- | --- |
| 28-day mortality | Path a (X → M)^b^ | 88.512 | 15.419 | 58.077 | 118.947 | <0.001 |
|  | Path b (M → Y)^c^ | 0.001092 | 0.000333 | 0.000437 | 0.001747 | 0.001 |
|  | Indirect effect^d^ | 0.0968 | 0.0342 | 0.0293 | 0.1643 | 0.005 |
|  | Direct effectd^e^ | 0.5236 | 0.1511 | 0.2241 | 0.8230 | 0.001 |
|  | Total effect^f^ | 0.6079 | 0.1500 | 0.3094 | 0.9064 | <0.001 |
|  | Proportion mediated^g^ | 15.92% | NA | 9.65% | 25.12% | 0.000 |
| 1-year mortality | Path a (X → M) | 88.51 | 15.42 | 58.08 | 118.95 | <0.001 |
|  | Path b (M → Y) | 0.000961 | 0.000332 | 0.000305 | 0.001618 | 0.004 |
|  | Indirect effect | 0.085 | 0.034 | 0.019 | 0.152 | 0.013 |
|  | Direct effect | 0.432 | 0.124 | 0.188 | 0.677 | 0.001 |
|  | Total effect | 0.496 | 0.121 | 0.257 | 0.734 | <0.001 |
|  | Proportion mediated | 17.10% | NA | 9.31% | 29.28% | 0.002 |

ΔNED, change in norepinephrine-equivalent dose; SE, standard error; CI, confidence interval; LL, lower limit; UL, upper limit; NA, not applicable.

^a^The pooled estimate and 95% CI were obtained by combining estimates from 5 imputed datasets using Rubin's rules.

^b^Path a represents the effect of the β-blocker group (X) on the change in norepinephrine-equivalent dose, ΔNED (M), with the Alternative AAD Group as the reference.

^c^Path b represents the effect of ΔNED (M) on mortality (Y), conditional on X.

^d^The indirect effect (a×b) and its 95% CI were derived using Rubin's rules from 5 imputed datasets.

^e^The direct effect represents the effect of the β-blocker group (X) on mortality (Y) not mediated through ΔNED (M).

^f^The total effect is the sum of the direct and indirect effects.

^g^The proportion mediated was calculated as the absolute value of the indirect effect divided by the total effect.

eFigure 1. Density Distribution of Propensity Scores


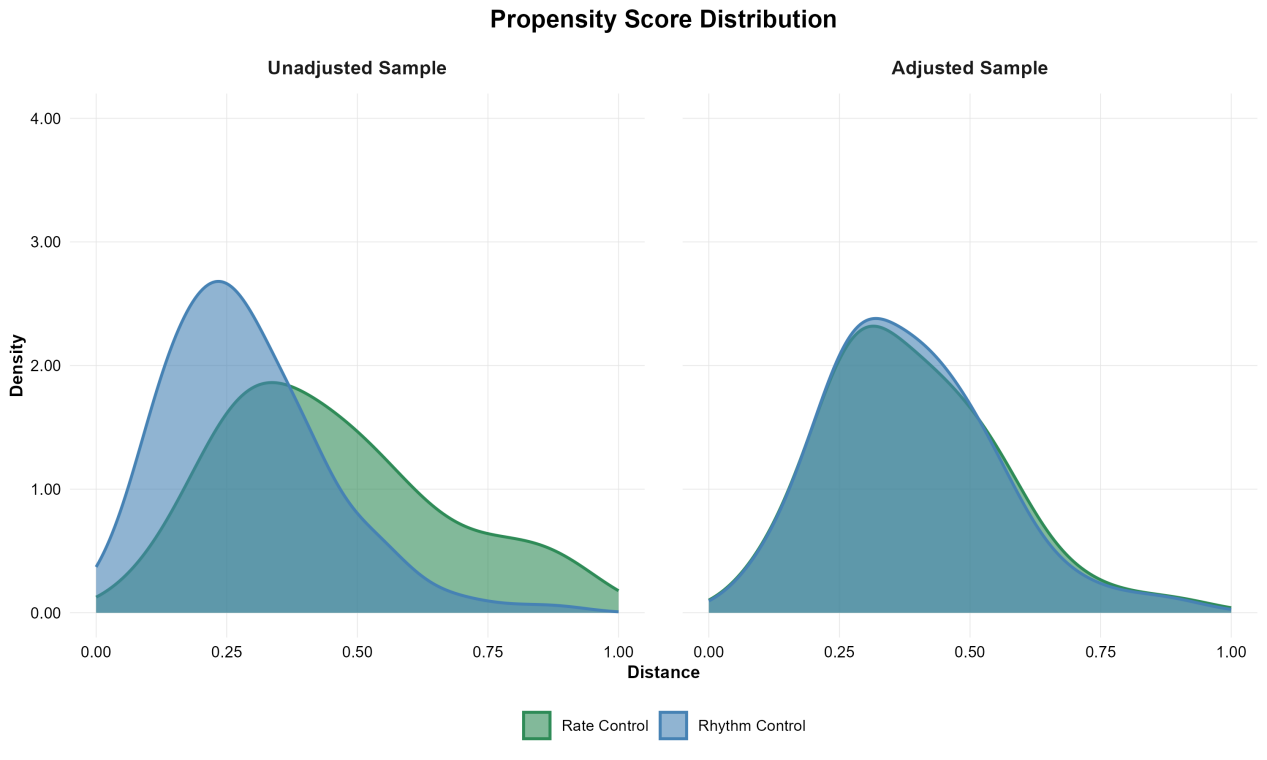


Density distributions of propensity scores for β-blocker group (green) and alternative antiarrhythmic drug group (blue) before (left) and after (right) matching. The increased overlap indicates improved covariate balance.

eFigure 2. Covariate Balance Plot


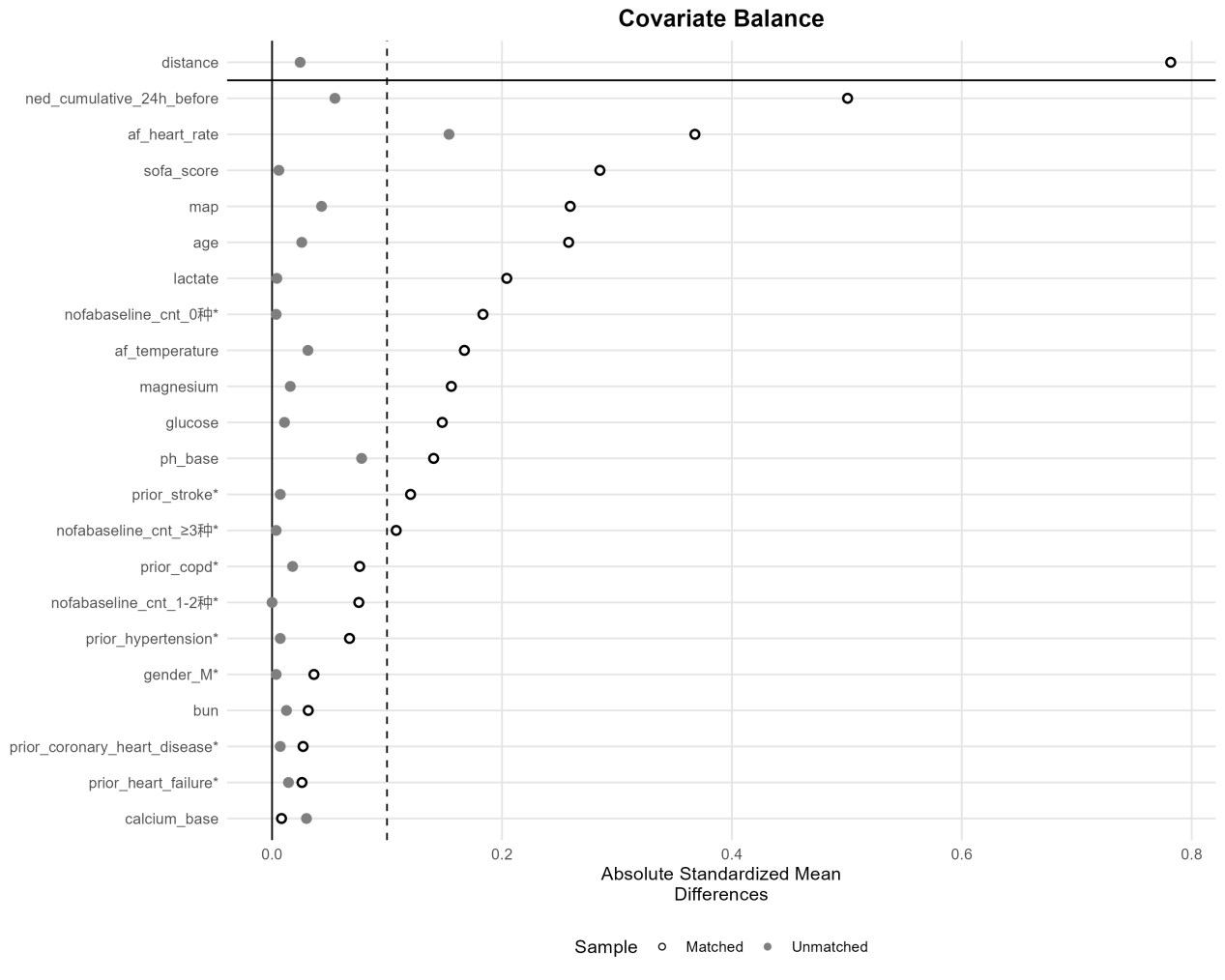


eFigure 3. Kaplan-Meier Curves for 28-Day and 1-Year Mortality by Treatment Strategy in the Original Cohort


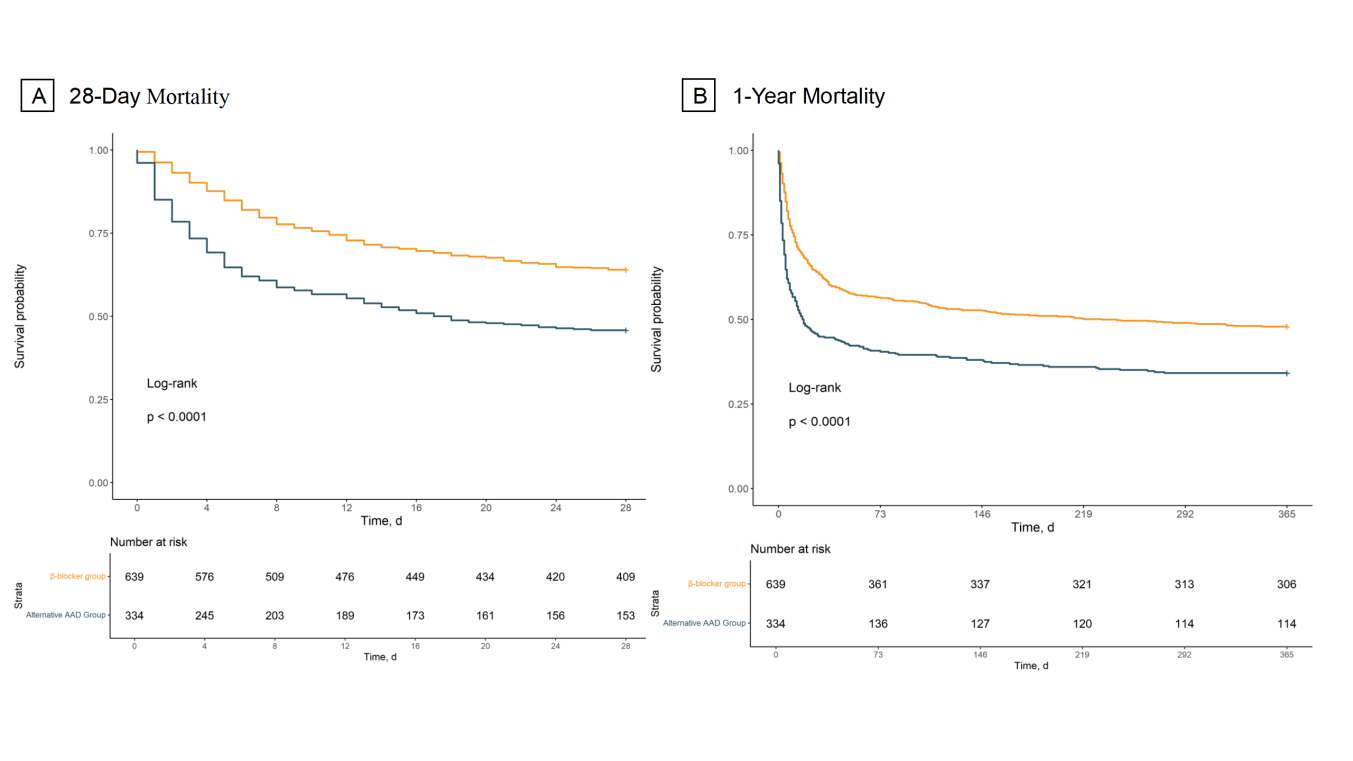


Data are from the Medical Information Mart for Intensive Care (MIMIC)-IV database;AAD, antiarrhythmic drug.

eFigure 4. Mediation Pathway Diagram and Bootstrap Mediation Effect Distribution


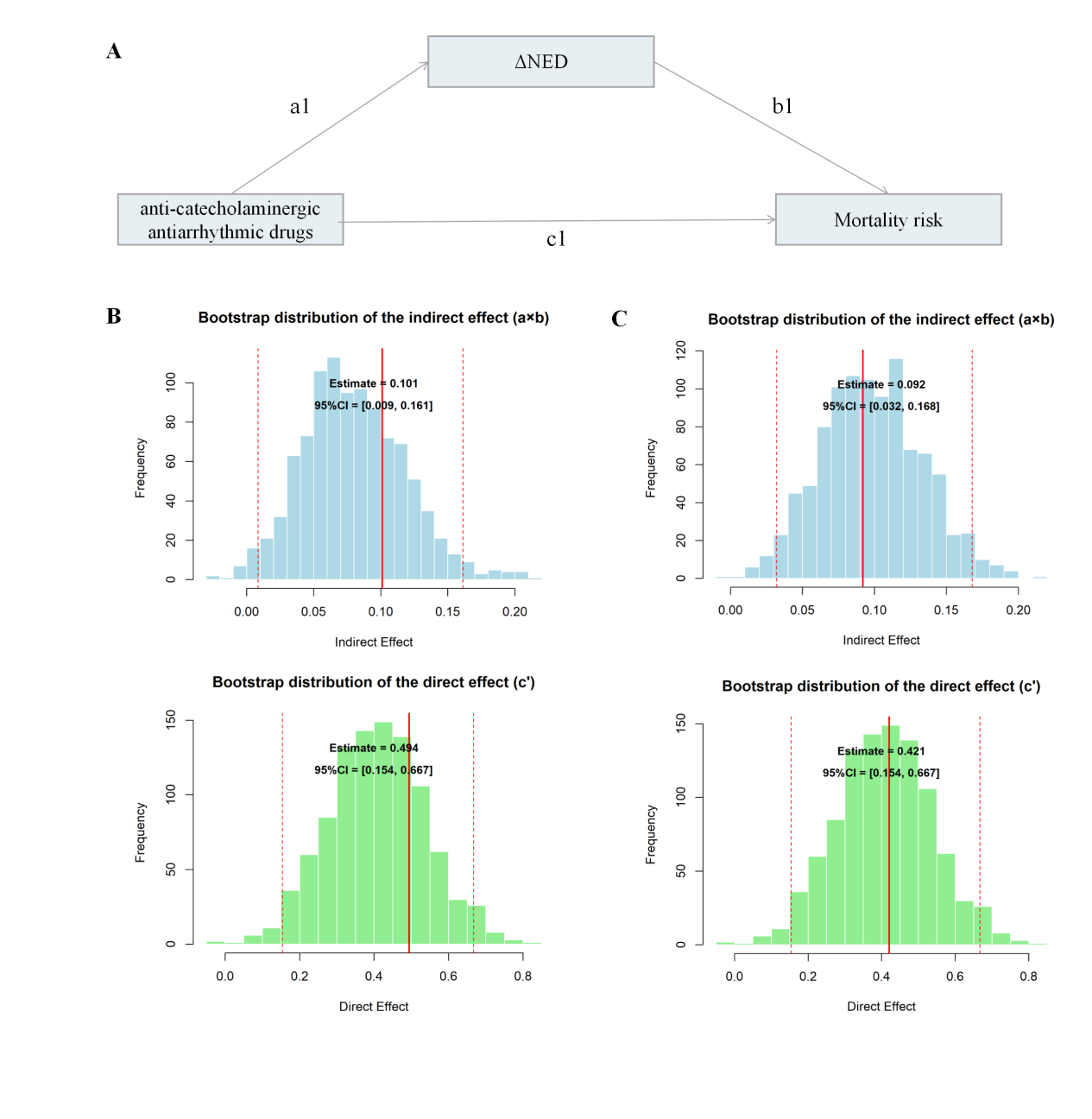


Bootstrap distributions of the indirect effect (a×b, upper panel) and direct effect (c′, lower panel) of anti-catecholaminergic anti-arrhythmic drugs on mortality risk via norepinephrine-equivalent dose (NED). Solid vertical lines indicate point estimates; dashed vertical lines denote 95 % bootstrap confidence intervals.
